# Failure of dihydroartemisinin-piperaquine treatment of uncomplicated *Plasmodium falciparum* malaria in a traveller coming from Ethiopia

**DOI:** 10.1186/s12936-016-1572-3

**Published:** 2016-11-03

**Authors:** Federico Gobbi, Dora Buonfrate, Michela Menegon, Gianluigi Lunardi, Andrea Angheben, Carlo Severini, Stefania Gori, Zeno Bisoffi

**Affiliations:** 1 Centre for Tropical Diseases, Hospital Sacro Cuore-Don Calabria, Via Sempreboni 5, Negrar, 37024 Verona, Italy; 2 Department of Infectious, Parasitic and Immunomediated Diseases (MIPI), Istituto Superiore di Sanità, Viale Regina Elena, 299, 00161 Rome, Italy

**Keywords:** Dihydroartemisinin-piperaquine (DHA-PPQ), Failure, *Plasmodium falciparum*, Recrudescence

## Abstract

**Background:**

Artemisinin combination therapy (ACT) is used worldwide as the first-line treatment against uncomplicated *Plasmodium falciparum* malaria. Despite the success of ACT in reducing the global burden of malaria, the emerging of resistance to artemisinin threatens its use.

**Case report:**

This report describes the first case of failure of dihydroartemisinin-piperaquine (DHA-PPQ) for the treatment of *P. falciparum* malaria diagnosed in Europe. It occurred in an Italian tourist returned from Ethiopia. She completely recovered after the DHA-PPQ treatment but 32 days after the end of therapy she had a recrudescence. The retrospective analysis indicated a correct DHA-PPQ absorption and genotyping demonstrated that the same *P. falciparum* strain was responsible for the both episodes.

**Conclusion:**

In consideration of the growing number of cases of resistance to ACT, it is important to consider a possible recrudescence, that can manifest also several weeks after treatment.

## Background

Artemisinin combination therapy (ACT) is used worldwide as the first-line treatment against uncomplicated falciparum malaria [1]. Dihydroartemisinin-piperaquine (DHA-PPQ) is characterized by a post-treatment prophylactic effect against re-infections that is longer than artemether-lumefantrine [2]. Despite the success of ACT in reducing the global burden of malaria, the emergence of resistance to artemisinin threatens its use. In Cambodia, failure of ACT is now frequently observed [3, 4]. The increase of treatment failures and parasite clearance times observed soon after the widespread introduction of DHA-PPQ suggests a rapid emergence of resistance to both artemisinin and piperaquine components. In Asia, treatment failures have been reported in Myanmar [5]. In South America, data on (good) efficacy of DHA-PPQ is based on only one trial, conducted in Peru between 2003 and 2005 [6]. In Africa, trials conducted in Burkina Faso [7], Kenya [8] and Angola [9] showed that DHA-PPQ was highly effective, with very rare cases of recrudescence invariably within 28 days.

## Case report

A 72-year-old Italian woman was admitted on 26 November, 2014 to the Centre for Tropical Diseases (CTD) of Negrar (Verona), for myalgias and arthralgias since 2 days, fever (up to 40 °C) and nausea since one day. She had visited Ethiopia (Omo River Valley) from 6 to 18 November 2014. She was vaccinated against yellow fever, hepatitis A and B, but had not taken any malaria chemoprophylaxis. Her travel history included Ethiopia, Niger, India, and Nambia, not South East Asia. Upon admission, her temperature was 38.3 °C, her weight 67.5 kg. Physical examination was unremarkable. The blood tests showed white blood cells (WBCs) 3.85 × 10^9^/L (normal range 5.2–12.4 × 10^9^/L), haemoglobin (Hb) 12.5 g/dL (normal range 14–18 × g/dL), platelets 46 × 10^9^/L (normal range 130–400 × 10^9^/L), C-reactive protein 135 mg/L (normal range 0–5 mg/L), procalcitonin 14 μg/L (normal range 0–0.5 μg/L). The quantitative buffy coat (QBC) test, antigen malarial test and blood smears resulted positive for *Plasmodium falciparum*, with a parasitaemia of 0.3% (14,600/μL). The patient was treated with DHA-PPQ 320/40 mg, three tablets/day for 3 days. The first day after treatment the parasitaemia dropped to 0.0023% (96/μL). After two days, the blood films and the QBC test resulted negative. Also, iv ceftriaxone 2 g/day was administrated for a left basal bronchopneumonia. The patient was discharged on 5 December, 2014.

At a follow-up visit on 23 December, the QBC test and blood smears were still negative. The blood tests showed WBC 7.98 × 10^9^/L, Hb 11.8 g/dL, platelets 233 × 10^9^/L.

She was re-admitted on 7 January, 2015 complaining of fever, nausea and vomiting that had started 7 days before (more than 4 weeks after anti-malaria treatment). The QBC test, antigen malarial test and blood smears all resulted positive again for falciparum malaria, with a parasitaemia of 0.4% (12,100/μL). WBCs were 5.5 × 10^9^/L, Hb 9.5 g/dL, platelets 96 × 10^9^/L. The patient was treated this time with atovaquone-proguanil 250/100 mg, four tablets/day for 3 days. The parasitaemia decreased to 0.36% (10,930/μL) 24 h after first dose of treatment, 0.16% (5017/μL) the second day, 0.0016% (46/μL) the third day. After 4 days, blood films resulted negative. The patient was discharged on 12 January, 2015.

At follow-up visits 28 and 56 days after the second malaria episode, QBC and blood smears resulted negative and the main laboratory findings were normal.

The malaria-PCR performed a posteriori on a blood specimen collected on 23 December, 2014 resulted positive. Serum DHA-PPQ concentrations were retrospectively evaluated on cryo-preserved (−80 °C) samples taken on different days, during and after the drug administration, coupled with tandem-mass spectrometry (Table 1). Limits of quantification for DHA and PPQ were 0.31 and 12.5 ng/mL, respectively. Pharmacokinetic data after the last dose of the three-day course of DHA and PPQ were comparable to data published by Nguyen et al. [10] indicating no defect in drug absorption. PPQ serum concentration on day 7 was calculated on the basis of the drug elimination half-life in this patient. Calculated PPQ concentrations on day 7 showed a serum level of 110 ng/mL (levels below 30 ng/mL have been associated to a higher risk of recurrence of malaria) [11].

**Table 1:** Serum concentrations of dihydroartemisinin-piperaquine

	Piperaquine (ng/mL)	Dihydroartemisinin (ng/nL)	Hours since last administration
Day 1	132	0.74	10
Day 2	282	0.34	15
Day 3	234	1.34	15
Day 4	205	N.v.	39
Day 5	131	–	63
Day 28	18	–	591
Day 7 (calculated)	110	–	111

**Fig. 1:**
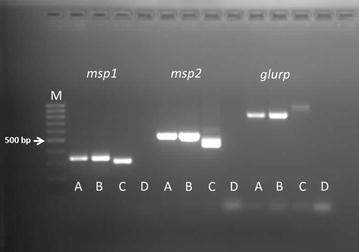
Genotyping of *Plasmodium falciparum* isolates responsible for the patient’s infection. *M* DNA ladder; *A* sample November 27, 2014; *B* sample January 7, 2015; *C* positive control; *D* negative control

Genotyping of *P*. *falciparum* isolate(s) responsible for the patient’s infection was performed by amplification of three polymorphic markers, the merozoite surface protein 1 (*msp1*), merozoite surface protein 2 (*msp2*), and glutamate-rich protein (*glurp*) genes. These genes show a length polymorphism, allowing the detection of multiple infections by different *P*. *falciparum* genotypes [12, 13]. Total genomic DNAs were extracted using PureLink Genomic DNA Kits-Invitrogen, from 200 µl of whole infected blood samples collected from the patient at the first (27 November, 2014) and second (7 January, 2015) hospital admittances. PCR amplified for *msp1/msp2* and for *glurp* as described by Wooden et al. [12] and Viriyakosol et al. [13], respectively. Genetic characterization of the *P*. *falciparum* isolates showed the presence of a single isolate responsible for the first episode and for the recurrence (Fig. 1). In order to investigate the resistance to the anti-malarial drugs of the isolate of *P. falciparum* infecting the patient, PCR amplification and sequencing were performed, in order to evaluate the presence of point mutations in the six molecular markers of *P*. *falciparum (PfK13, Pfcrt, Pfmdr1, Pfdhfr, Pfdhps* and *PfCytB* genes*)* linked to resistance to the artemisinin derivatives, quinolines, antifolates-cycloguanil and atovaquone. The polymorphism of the propeller domain of the *Pfk13* gene was assessed as described by Taylor et al. [14]. Analysis of *Pfcrt*/*Pfdhps* and *Pfmdr1* and genes was performed as previously described in Menegon et al. [15] and Duraisingh et al. [16], respectively. A fragment of *Pfdhfr* gene spanning codons 51–108 was analysed as described by Palmieri et al. [17]. The presence of point mutation in PfCytB gene was evaluated as described in Korsinczky et al. [18]. All PCR products were sent to Eurofins Genomics Company (Germany) for sequencing. The obtained sequences were compiled and analysed by Accelrys DS Gene software. The results are summarized in Table 2. Analysis of polymorphism of the *P*. *falciparum* isolates showed the presence of mutations in *Pfcrt* and *Pfmdr1* genes linked to resistance to the quinolines and mutation in *Pfdhfr* and *Pfdhps* genes correlated to antifolate/cycloguanil resistance. No mutations associated with artemisinin resistance and atovaquone were detected.

**Table 2:** Analysis of the molecular markers of *P. falciparum* linked to drug resistance

Specific point mutations in *Pfcrt* and *Pfmdr1* gene are linked to quinoline-based antimalarial resistance; *PfK13* is the molecular markers for artemisinin resistance; mutation I258M and Y268F/S in *PfCytB* gene are linked to atovaquone resistance; mutations in *Pfdhfr/Pfdhps* genes are responsible for *P. falciparum* resistance to antifolate-cycloguanil

## Conclusion

This is the first case of failure of DHA-PPQ reported in Europe. The patient returned from Ethiopia, a country where DHA-PPQ failures have not been reported before. In this case, the rapid response to DHA-PPQ and the lack of mutations in the *PfK13* gene suggest the involvement of an artemisinin-sensitive strain. Although it was not possible to analyse a specific molecular marker of resistance to PPQ (a newly identified gene, PFE1085w is presumably associated to resistance to this drug) [19], the combination of results obtained from molecular and pharmacokinetic analyses and the clinical characteristics support that the strain was resistant to the PPQ component.

In the last 2 years (July 2014 to June 2016) DHA-P was administered to 36 patients attended at the CTD for falciparum malaria, observing no other failure. These data are in agreement with the literature. The efficacy of DHA-PPQ has been found very high, particularly in the African continent. There was a relevant delay between the onset of symptoms and the second diagnosis because the index of suspicion was low due to the negative laboratory tests performed at the 28-day follow-up visit. In consideration of the growing number of cases of resistance to ACT, it is important to consider a possible recrudescence, which can manifest several weeks after treatment.
